# ISG15 Deficiency Enhances HIV-1 Infection by Accumulating Misfolded p53

**DOI:** 10.1128/mBio.01342-19

**Published:** 2019-08-27

**Authors:** Edmund Osei Kuffour, Renate König, Dieter Häussinger, Wolfgang A. Schulz, Carsten Münk

**Affiliations:** a Clinic for Gastroenterology, Hepatology and Infectious Diseases, Medical Faculty, Heinrich-Heine University Düsseldorf, Düsseldorf, Germany; b Host-Pathogen Interactions, Paul-Ehrlich-Institut, Langen, Germany; c Immunity and Pathogenesis Program, Sanford Burnham Prebys Medical Discovery Institute, La Jolla, California, USA; d Department of Urology, Medical Faculty, Heinrich-Heine University Düsseldorf, Düsseldorf, Germany; Columbia University/HHMI

**Keywords:** HIV-1, THP-1, USP18, p21, p53

## Abstract

Macrophages and dendritic cells dominate early immune responses to lentiviruses. HIV-1 sensing by pathogen recognition receptors induces signaling cascades that culminate in type I alpha/beta interferon (IFN-α/β) induction. IFN-α/β signals back via the IFN-α/β receptors, inducing a plethora of IFN-stimulated gene (ISGs), including ISG15, p53, and p21^Cip1^. p21 inhibits HIV-1 replication by inactivating the deoxynucleoside triphosphate (dNTP) biosynthesis pathway and activating the restriction factor SAMHD1. p21 is induced by functional p53. ISG15-specific isopeptidase USP18 negatively regulates IFN signaling. We showed previously that USP18 contributes to HIV-1 replication by abrogating p21 antiviral function. Here, we demonstrate a mechanism by which USP18 mediates p21 downregulation in myeloid cells. USP18, by its protease activity, accumulates misfolded p53, which requires ISG15 for its degradation. Depletion of ISG15 causes accumulation of misfolded dominant negative p53, which enhances HIV-1 replication. This work clarifies the function and consequences of p53 modification by ISG15 and implicates USP18 in HIV-1 infection and potentially in carcinogenesis.

## Abstract (precis)

HIV-1 has evolved many strategies to circumvent the host’s antiviral innate immune responses and establishes disseminated infection; the molecular mechanisms of these strategies are not entirely clear. We showed previously that USP18 contributes to HIV-1 replication by abrogating p21 antiviral function. Here, we demonstrate a mechanism by which USP18 mediates p21 downregulation in myeloid cells. USP18, by its protease activity, accumulates misfolded p53, which requires ISG15 for clearance. Depletion of ISG15 causes accumulation of misfolded dominant negative p53, which supports HIV-1 replication. This work clarifies the function and consequences of p53 modification by ISG15 and implicates USP18 in HIV-1 infection and potentially in carcinogenesis.

## INTRODUCTION

Macrophages and dendritic cells possess germ line-encoded pathogen recognition receptors (PRRs) that recognize conserved pathogen-associated molecular patterns (PAMPs) of human immunodeficiency virus type 1 (HIV-1) (1–10). The host PRR and HIV-1 PAMP interaction triggers innate immune signaling in the infected cells, culminating in the production of type I and III interferons (IFNs), including alpha/beta interferon (IFN-α/β). IFN-α/β signals back via the IFN-α receptor 1 and 2, driving more IFN-α/β production and induction of IFN-stimulated genes (ISGs), which help to block the replication and spread of the virus (4, 10). Apart from ISGs, other intrinsic antiviral factors are also produced or activated. Among them are ISG15, p21^Waf1/Cip1/Sdi1^ (here, p21), and the tumor suppressor p53 (*TP53*) (11–26).

p21 is a major downstream effector of p53. As a cyclin-dependent kinase (CDK) inhibitor, p21 mediates cell cycle arrest, DNA repair, senescence, and, in certain instances, cell death by apoptosis (27). p21 is induced following HIV-1 infection, and its expression inhibits HIV-1 in monocyte-derived macrophages (MDMs) and dendritic cells (MDDCs) (15, 27–37). p21 affects HIV-1 replication by regulating key enzymes involved in *de novo* deoxynucleoside triphosphate (dNTP) biosynthesis (33, 34). It inhibits HIV-1 replication by blocking transcriptional activation of the R2 subunit of ribonucleotide reductase (RNR2, also known as RRM2) by the host transcription factor E2F1 (33, 34). p21 further blocks HIV-1 replication by promoting dephosphorylation and activation of SAMHD1 restriction function (38–43) and by inhibiting CDK2-dependent phosphorylation of the HIV-1 reverse transcriptase (44). Experimental downregulation of p21 results in increased HIV-1 infection (45). The transcription, expression, and activity of p21 are regulated via p53-dependent and -independent pathways (35, 46–49). Under stress conditions, such as DNA damage or viral infection, p21 is highly upregulated, likely mediated by induction by activated p53 and other pathways (27, 35, 47, 50, 51). p53 expression and activity are also regulated by several posttranslational modifications, including ubiquitination, acetylation, phosphorylation, and ISGylation, all of which likely impact p21 induction and function. Posttranslational modification of p53 by ISG15 appears critically important for the regulation of p53 transactivation function; however, the mechanism of the ISG15-dependent regulation of p53 function may differ depending upon the cellular context (46, 48, 49, 52). Single point mutations, deletion, and rearrangement of the p53 gene affect p21 transcription and thus potentially impact HIV-1 infection and replication. Indeed, the absence of functional p53 decreases p21 expression and correlates significantly with the enhancement of HIV-1 infection and replication at the reverse transcription step (13, 14). Moreover, p53 itself is activated by HIV-1 infection, and its expression likely inhibits HIV-1 long terminal repeat (LTR) promoter activity (16, 53–56).

IFN-inducible ubiquitin-like specific protease USP18 (UBP43) negatively regulates type I and III IFN signaling pathways (57–59). USP18 targets ISG15 and cleaves it from its conjugated proteins (58–61). By interacting with IFNAR2, USP18 blocks IFN signaling by disrupting IFNAR2-JAK1 (Janus-activated kinase 1) binding in an isopeptidase-independent manner (59, 62, 63). In the absence of free ISG15, USP18 is targeted for ubiquitination and proteasomal degradation by SKP2 (64). USP18 depletion by experimental knockout enhances JAK/STAT (signal transducer and activator of transcription) signaling and increases ISGs, resulting in upregulated levels of protein ISGylation (59, 65–68).

We recently demonstrated that USP18 is HIV-1 inducible and that its expression enhances HIV-1 replication. The enhanced HIV-1 replication was mediated by downregulation of p21, which correlated with increased dNTP levels and phosphorylation of the inactive form of SAMHD1 (69). Here, we investigated the molecular mechanisms behind the USP18-mediated downregulation of p21 and its resultant elevation of dNTP levels and increased phosphorylated SAMHD1 in the myeloid THP-1 and BlaER1 cells.

## RESULTS

### USP18 relieves p21 repression of E2F1 and *de novo* dNTP biosynthesis pathway.

**FIG 1:**
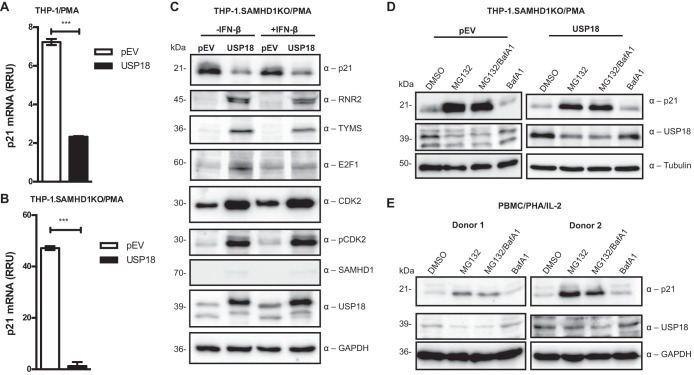
USP18 relieves p21 repression of E2F1 and *de novo* dNTP biosynthesis. (A) Total RNA from PMA-differentiated THP-1 cells expressing USP18 and vector controls (pEV) were isolated and quantified for p21 mRNA expression using quantitative RT-PCR (qRT-PCR) and normalized to HPRT1 (relative mRNA unit [RRU] = 7 and 2 for THP-1.USP18 and control cells, respectively). (B) Total RNA from PMA-differentiated SAMHD1KO THP-1 cells expressing USP18 and controls were isolated and quantified for p21 mRNA expression using qRT-PCR and normalized to HPRT1 (RRU = 47 and 2 for SAMHD1KO THP-1.USP18 and THP-1.pEV, respectively). (C) PMA-differentiated SAMHD1KO THP-1.USP18 and pEV cells were treated with or without IFN-β (1,000 U/ml). Twenty-four hours posttreatment, the cells were lysed and immunoblotted for p21, RNR2, TYMS, and E2F1 expression. Furthermore, total and phosphorylated CDK2, SAMHD1, USP18, and GAPDH as loading control were detected, using the respective antibodies. (D) PMA-differentiated SAMHD1KO THP-1 cells expressing USP18 and its vector controls were treated with DMSO, MG132 (5 μM), bafilomycin (BafA1; 10 nM), or both. Sixteen hours posttreatment, the cells were lysed and immunoblotted for p21, USP18, and tubulin as a loading control. (E) Activated PBMCs from two different donors were treated with DMSO, MG132, BafA1, or both and immunoblotted for p21, USP18, and GAPDH as a loading control. The average *C_T_* from two independent experiments expressed as mean ± SD was compared between the groups in panels A and B by Student's *t* tests. ***, *P* < 0.05. Each panel is representative of at least two independent experiments.

To understand the molecular mechanisms behind USP18-mediated downregulation of p21, we investigated p21 mRNA and protein expression (Fig. 1A and B) as well as downstream effector proteins of p21 in phorbol myristate acetate (PMA)-differentiated wild-type and SAMHD1 knockout (KO) THP-1.USP18 cells (Fig. 1C). Interestingly, p21 expression was downregulated not only at the protein level but also strongly at the transcriptional level in wild-type THP-1.USP18 cells compared to that in vector controls (pEV) (Fig. 1A and B). p21 mRNA levels were reduced by approximately 3-fold (Fig. 1A), and this effect was even more prominent (>30-fold) in the absence of SAMHD1 in the THP-1.USP18 cells (Fig. 1B). Considering that the SAMHD1KO cells exhibited significantly low p21 mRNA and to avoid the pleotropic effect of viral protein VPX in our infection assays (70), we explored further the mechanism of USP18-mediated downregulation of p21 in the SAMHD1KO THP-1 cells. Interestingly, key enzymes of *de novo* dNTP biosynthesis were all significantly upregulated in SAMHD1KO THP-1.USP18 cells compared to that in the control cells (Fig. 1C). Downregulation of p21 expression by USP18 correlated strongly with upregulated total and phosphorylated CDK2, RNR2, E2F1, and TYMS in SAMHD1KO THP-1.USP18 cells compared to levels in their controls (Fig. 1C). The presence of IFN-β (1,000 U/ml) did not alleviate this effect, except for reducing slightly the level of E2F1 (Fig. 1C). p21 downregulation was however rescued by the proteasome inhibitor MG132 (Fig. 1D) and in activated primary peripheral blood mononuclear cells (PBMCs) (Fig. 1E). In contrast, expression of USP18 was reduced in both SAMHD1KO THP-1 and primary cells by MG132 treatment (Fig. 1D and E). Lysosomal inhibitor bafilomycin had no effect on p21 upregulation (Fig. 1D and E).

### USP18 stabilizes p53 expression in differentiated myeloid THP-1 cells.

**FIG 2:**
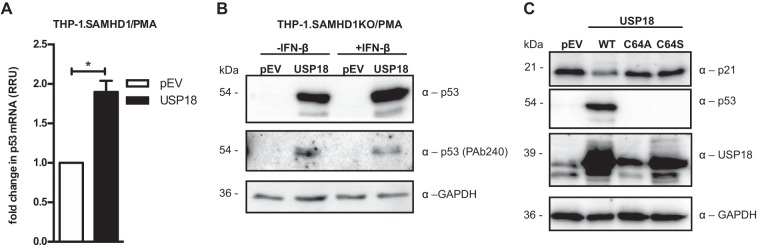
USP18 stabilizes p53 expression in differentiated THP-1 cells. (A) p53 mRNA expression in the SAMHD1KO THP-1.USP18 and control cells was quantified by qRT-PCR, normalized to HPRT1 (RRU = 0.15 and 0.34, respectively). (B) PMA-differentiated SAMHD1KO THP-1.USP18 and pEV cells were treated with or without IFN-β (1,000 U/ml). Twenty-four hours posttreatment, the cells were lysed and immunoblotted for p53 (antibody Ab-6) and dominant negative misfolded p53 (antibody PAb240), with GAPDH as a loading control. (C) PMA-differentiated stable SAMHD1KO THP-1 cells expressing wild-type USP18, active-site mutants (C64A and C64S), or the pEV were immunoblotted for p21, p53, USP18, and GAPDH using the indicated antibodies. Each panel is representative of at least two independent experiments. *, *P* < 0.05.

Considering that SAMHD1KO THP-1.USP18 cells exhibited significantly low p21 mRNA expression, we tested for mRNA and protein expression of p53, a promoter of p21 (49, 71). Interestingly, we observed a slight elevation of p53 mRNA in the SAMHD1KO THP-1.USP18 cells (approximately 2-fold) (Fig. 2A) and rather high expression of p53 protein compared to that of the controls (Fig. 2B). A monoclonal antibody (PAb240) that recognizes an epitope exposed by activating mutations or denaturation (72) detected misfolded p53 in the SAMHD1KO THP-1.USP18 cells but not in the pEV (Fig. 2B). Because p53 transcription is thought to be boosted by type I IFN (17, 19) and USP18 is a negative regulator of type I IFN, we tested for p53 expression in the SAMHD1KO THP-1.USP18 cells and pEV, with and without IFN-β treatment. We indeed observed strong expression of p53 in the SAMHD1KO THP-1.USP18 cells, which was slightly increased by IFN-β, but no p53 expression in the pEV (Fig. 2B). Despite the high expression of p53 in the SAMHD1KO THP-1.USP18 cells, p21 expression remained low (Fig. 2C). Conversely, cells expressing active-site mutants of USP18 (C64A or C64S) showed elevated p21 but lacked p53 expression compared to that in wild-type USP18 cells (Fig. 2C). This suggests that the p53 protein accumulating in USP18-expressing cells is not a wild-type p53.

### p53 is modified by ISG15.

**FIG 3:**
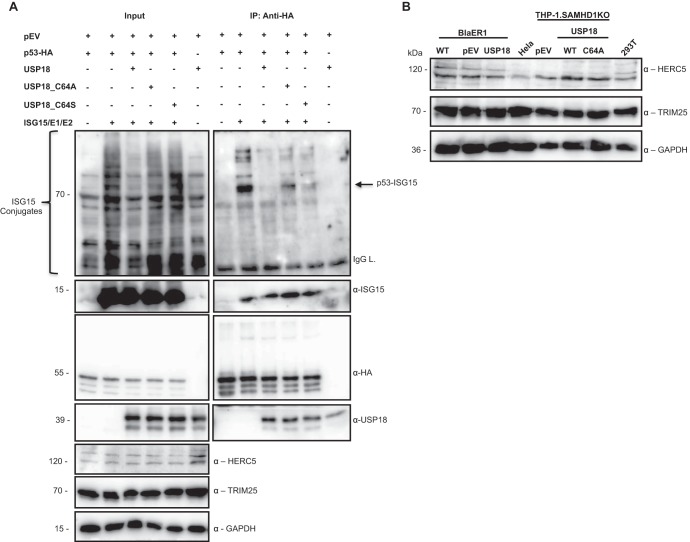
p53 is modified by ISG15. (A) 293A cells were singly transfected or cotransfected with expression plasmids for HA-tagged p53 (p53-HA), p53-HA and USP18, or p53-HA and active-site mutants of USP18 (C64A or C64S) in the presence of expression plasmids for ISG15 and its activating enzyme E1 (UBE1L) and conjugating enzyme E2 (UBCH8). Single transfections were supplemented with pLOC empty vector (pEV). Forty-eight hours after transfection, the cells were harvested, immunoprecipitated for p53 using anti-HA affinity matrix, and subsequently immunoblotted for free ISG15 and its conjugates, HA, USP18, endogenous HERC5, and TRIM25 (EFP) using respective antibodies. IgG L denotes immunoglobulin G light chain of the HA antibody. (B) Protein lysates from wild-type, vector control, and USP18-expressing BlaER1 cells and PMA-differentiated SAMHD1KO THP-1 cells expressing vector control, USP18, and its active-site mutant C64A were immunoblotted for endogenous expression of HERC5, TRIM25, and GAPDH. Lysates from HeLa and 293T cells were included as positive controls for endogenous TRIM25 and HERC5 expression, respectively. The panels are representative of at least 3 independent experiments.

Considering that the active-site mutants of USP18 failed to accumulate p53, we wondered whether p53 is modified by ISG15. Previously, two independent reports suggested ISG15-dependent positive regulation of p53, albeit by different mechanisms with differing E3 ligases mediating ISG15 modification of p53 (46, 49). To confirm the modification of p53 by ISG15, we coexpressed p53 and ISG15 with its activating enzyme E1 (UBE1L) and conjugating enzyme E2 (UBCH8), in the presence of either USP18 or its mutants, in 293A cells which lack the SV40 large T antigen. Considering that two different E3 ligases (HERC5 and TRIM25) have both been shown to mediate p53 ligation to ISG15 (46, 49), we relied on the endogenous expression of these E3 ligases, consistent with Park et al. (49) for p53 modification in our overexpression system (46). Immunoprecipitation of p53 and immunoblotting for ISG15 showed that ISG15 was indeed covalently linked to p53 (Fig. 3A), indicating that the expression levels of the endogenous E3 ligases were sufficient to confer this modification. Indeed, both HERC5 and TRIM25 were robustly expressed in our cell models (Fig. 3B). Furthermore, the ISG15 modification of p53 was abrogated by USP18. Moreover, modification of p53 by ISG15 was partially rescued by a mutation in the active site of USP18 (Fig. 3A).

### USP18 accumulates misfolded dominant negative p53.

**FIG 4:**
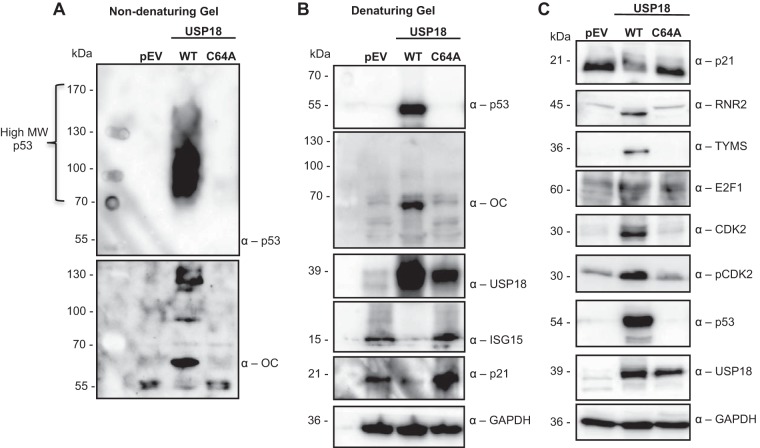
USP18 accumulates misfolded dominant negative p53. (A) Protein lysates from PMA-differentiated SAMHD1KO THP-1 cells expressing USP18 and its active-site mutant C64A were immunoblotted for high-molecular-weight (HMW) p53 and amyloid fibrils using anti-p53 antibody and anti-amyloid antibody (OC) after nondenaturing gel electrophoresis. pEV, control cells with empty vector. (B) Protein lysates from PMA-differentiated SAMHD1KO THP-1 cells expressing USP18 and its active-site mutant C64A were immunoblotted for p53, amyloid fibrils (anti-OC), USP18, ISG15, p21, and GAPDH using the indicated antibodies after sodium dodecyl sulfate (SDS)-mediated denaturing gel electrophoresis. pEV, control cells with empty vector. (C) Protein lysates from PMA-differentiated SAMHD1KO THP-1 cells expressing pEV, USP18, and its active-site mutant C64A were immunoblotted for p21, RNR2, TYMS, E2F1, total and phosphorylated CDK2, p53, USP18, and GAPDH using the respective antibodies after SDS-mediated denaturing gel electrophoresis. Each panel is representative of at least 2 independent experiments.

Considering that the USP18-mediated p53 accumulation in the SAMHD1KO THP-1 cells failed to induce p21 expression, we asked whether the accumulating p53 could be a misfolded dominant negative protein (46, 72–82). We therefore tested for p53 aggregation and amyloid-like fibrils in the PMA-differentiated SAMHD1KO THP-1.pEV and USP18 or its C64A mutant under nondenaturing and denaturing conditions (76). Indeed, immunoblots for p53 and amyloid fibrils after nondenaturing gel electrophoresis revealed high-molecular-weight p53 that appeared as a smear in the blot in the SAMHD1KO THP-1.USP18 cells (Fig. 4A). In contrast, p53 appeared at the size expected for a monomer in SDS-denaturing gel electrophoresis (Fig. 4B). Interestingly, the USP18-mediated p53 aggregation correlated significantly with low ISG15 and p21 expression in the PMA-differentiated SAMHD1KO THP-1 cells (Fig. 4B). Furthermore, the decreased p21 expression in the USP18 cells correlated significantly with upregulation of total and phosphorylated CDK2, E2F1, RNR2, and TYMS (Fig. 4C). These effects were reversed by a mutation in the active site of USP18 (Fig. 4C).

### ISGylation is required for *in vivo* clearance of misfolded dominant negative p53.

**FIG 5:**
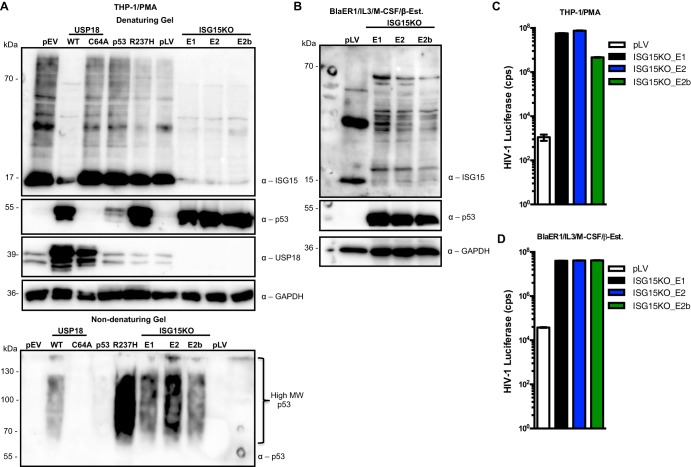
ISGylation is required for *in vivo* clearance of misfolded dominant negative p53. (A) Protein lysates from PMA-differentiated SAMHD1KO THP-1 cells expressing USP18 and its active-site mutant C64A, wild-type p53 and its single-amino-acid mutant (R273H), pLentiCRISPR empty vector control (pLV), and ISG15KO wild-type THP-1 cells were immunoblotted for ISG15, monomeric p53, USP18, and GAPDH using the respective antibodies after SDS-mediated denaturing gel electrophoresis. In a similar experiment, the lysates were immunoblotted for the oligomeric form (HMW) of p53 using the indicated antibody after nondenaturing gel electrophoresis. (B) Protein lysates from transdifferentiated ISG15KO BlaER1 cells were immunoblotted for ISG15, monomeric p53, USP18, and GAPDH using the respective antibodies after SDS-mediated denaturing gel electrophoresis. pLV, cells with empty CRISPR vector. (C) PMA-differentiated ISG15KO and control THP-1 (pLV) cells were transduced with HIV-1 luciferase reporter virus. Forty-eight hours postransduction, the cells were measured for luciferase activity. E1, sgRNA targeting exon 1; E2, sgRNA targeting exon 2; E2b, different sgRNA targeting exon 2. (D) Transdifferentiated ISG15KO BlaER1 and control cells (pLV) were transduced with HIV-1 luciferase reporter virus. Forty-eight hours postransduction, the cells were measured for luciferase activity. Each panel is representative of at least 2 independent experiments.

To ascertain that ISGylation is important for the clearance of misfolded dominant negative p53 in myeloid cells (46, 48), we depleted THP-1 cells of ISG15 and tested for p53 aggregation and amyloid fibrils. As a control, we included PMA-differentiated THP-1 cells expressing wild-type p53 or a well-characterized single-amino-acid mutant of p53 (R273H), which has been shown to form protein aggregates in a misfolded conformation (73, 76–79, 81–84). Intriguingly, the absence of ISG15 led to accumulation of high-molecular-weight p53 and amyloid fibrils, reminiscent of the phenotype exhibited by the R273H mutant p53 or following USP18 expression (Fig. 5A). Furthermore, PMA-differentiated THP-1.ISG15KO cells lost the expression of USP18, underlining the requirement of ISG15 for stabilizing USP18 (85) (Fig. 5A). This observed phenotype was not exclusive to the THP-1 cells but likely applies to all myeloid cells, as BlaER1.ISG15KO cells also exhibited accumulated p53 expression (Fig. 5B). As a consequence of the accumulated p53 in the PMA-differentiated THP-1.ISG15KO and transdifferentiated BlaER1.ISG15KO cells, HIV-1 replication was enhanced (Fig. 5C and D).

### HIV-1 induces p53 and “gain-of-function” mutant p53 supports HIV-1 replication.

**FIG 6:**
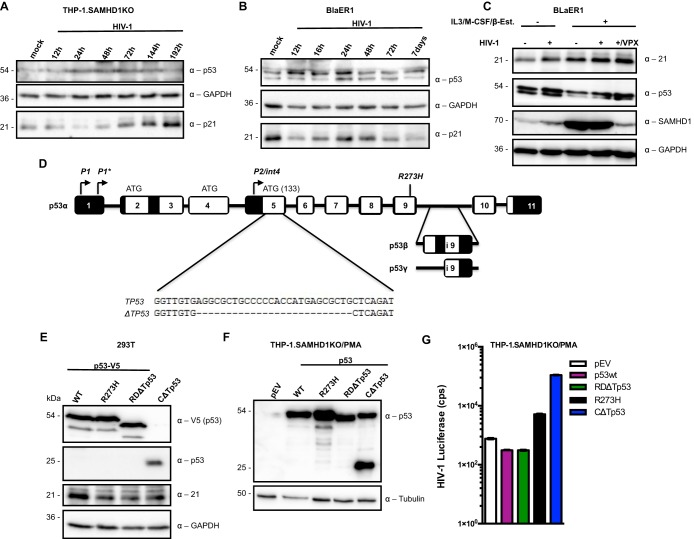
HIV-1 induces p53 and the “gain-of-function” p53 mutants promote HIV-1 replication. (A) SAMHD1KO THP-1 cells were transduced with HIV-1 (multiplicity of infection [MOI] = 2). At the indicated time points, cells were harvested, lysed, and immunoblotted for p53 and p21, with GAPDH as a loading control, using the indicated antibodies. (B) BlaER1 cells were transduced with HIV-1 (MOI = 2). At the indicated time points, cells were harvested, lysed, and immunoblotted for p53, p21, and GAPDH using the indicated antibodies. (C) Undifferentiated and transdifferentiated BlaER1 cells were transduced with HIV-1, with and without VPX (MOI = 2). Twenty-four hours postransduction, the cells were harvested, lysed, and immunoblotted for p21, p53, SAMHD1, and GAPDH using the indicated antibodies. (D) Intron exon structure of the p53 gene. THP-1 cells have one intact p53 gene (*TP53*) and one allele with a 26 bp deletion (Δ*TP53*) as detected using genomic DNA. P, promoter; R273H: point mutation found in many cancers; p53β and p53γ, alternative exons. (E) V5-tagged wild-type p53, single-amino-acid (R273H) mutant p53, RD domain mutant (RDΔTp53), and an untagged C-terminal truncation mutant (CΔTp53) of p53 were expressed in 293T cells. Protein lysates from these cells were immunoblotted for anti-V5, 25-kDa truncation mutant p53, p21, and GAPDH as a loading control. (F) pLOC plasmids expressing wild-type p53, R273H, RDΔTp53, or CΔTp53 were stably expressed in the THP-1 cells. These cell lines were subsequently differentiated by 25 ng/ml of PMA and immunoblotted for p53 and GAPDH as a loading control. (G) SAMHD1KO THP-1 cells expressing wild-type p53 and its mutants (R273H, RDΔTp53, or CΔTp53) were transduced with HIV-1 luciferase reporter virus. Forty-eight hours after transduction, luciferase activity was measured. Each panel is representative of at least 2 independent experiments.

Two independent studies suggested that HIV-1 induces p53 in lymphocytes (51, 86). However, p53 induction by HIV-1 in myeloid cells has not been reported to date. We therefore transduced SAMHD1KO THP-1 cells with HIV-1 and investigated p53 expression at different time points. We observed p53 induction 24 h after transduction, which persisted until later time points when p53 disappeared gradually and p21 became induced (Fig. 6A). To confirm this observation in a different myeloid cell, we transduced undifferentiated BlaER1 cells (Fig. 6B). Here, p53 expression appeared as early as 12 h postransduction and persisted until 48 h when the signal weakened, correlating with p21 induction at 24 h and its disappearance after 72 h (Fig. 6B). In a related experiment in undifferentiated and transdifferentiated BlaER1 cells, transient induction of p53 was observed 24 h after HIV-1 infection in the presence and absence of VPX, which correlated significantly with p21 induction with high expression in the presence of VPX from HIV-2, which degrades SAMHD1 (87–90) (Fig. 6C). THP-1 cells have two different p53 alleles, one wild type and another allele containing a 26-bp deletion in exon 5 (Fig. 6D). The latter variant (CΔTp53) was cloned and expressed in 293T cells in comparison to wild-type p53, a single-amino-acid inactive mutant (R273H) (73, 75, 79, 82, 91, 92), and a C-terminal DNA-binding regulatory domain (RD) deletion mutant (RDΔTp53), which retains an intact DNA-binding domain as an additional control. The 26-bp deletion causes a frameshift resulting in an approximately 25-kDa truncated protein (Fig. 6E and F). We next expressed the wild-type p53 and its mutants in the SAMHD1KO THP-1 cells and checked for p53. All cells with mutant p53 maintained stable p53 expression and remained viable. On the contrary, the wild-type SAMHD1KO THP-1.p53 cells exhibited reduced p53 expression, likely because of reduced viability (Fig. 6F). Interesting, the 25-kDa mutant p53 in the THP-1 cells elevated the expression of the 53-kDa protein (Fig. 6F). To analyze whether mutant p53 support HIV-1 replication, SAMHD1KO THP-1.p53 and its mutants were transduced with HIV-1 reporter virus. The wild type and the RD domain deletion mutant p53 reduced HIV-1 infection; however, cells expressing the single-amino-acid variant (R273H) or the 26-bp deletion mutant p53 from the THP-1 cells were highly susceptible to HIV-1 infection (Fig. 6G).

## DISCUSSION

Mounting evidence suggests p21 is an important host intrinsic innate immune resistance factor against lentiviruses (13, 29, 31–34, 44, 45). The synthesis of p21 mRNA and protein is under transcriptional regulation by p53 as well as by p53-independent pathways (27, 35, 47).

Under stressed conditions, including retroviral infection and genotoxic-induced DNA double-strand breaks, DNA-dependent protein kinase (DNA-PK) and ATM signal a DNA damage response (51, 86, 93–98). This process causes the stabilization and posttranslational modifications of p53 by phosphorylation and acetylation, leading to p53 activation (46, 49, 51, 93, 94, 99). The increased expression and activation of p53 proteins transactivates CDKNIA transcription, leading to p21 mRNA and protein expression and inducing cell cycle arrest and repair of the damaged DNA (19, 91, 100, 101). Following repair of DNA damage, p53 is likely targeted for the proteasome either by MDM2-mediated ubiquitin-dependent degradation (19, 27, 49, 91, 100, 101) or by ISGylation-dependent degradation (19, 46, 48). Dysregulation of p53 is a hallmark of many tumors likely caused by mutations, deletions, and rearrangements in the p53 gene (27, 74, 79, 92, 100–105). Mutant p53 lacks the transactivation function and can also dysregulate the wild-type p53 (46, 73, 74, 79, 91, 92, 103–106).

p53 and its downstream effector gene, p21, are both HIV-1 and type I IFN inducible (11, 15, 17, 19, 37, 51, 69, 86, 107). Induction and activation of p53 by HIV-1 possibly occur at the level of the viral cDNA integration into the host genome. The HIV-1-integrase-mediated double-strand break likely signals a DNA damage response mediated by the activation of DNA-PK and ATM (51, 86, 108, 109).

Two different mechanisms have been proposed to underlie the ISG15-dependent regulation of p53 function. One model suggests that ISG15 conjugation to newly synthesized unstructured p53 is required for the degradation of misfolded dominant negative p53 by the 20S proteasome, a mechanism that preserves p53-mediated biological function (46, 48). Alternatively, it is discussed that under cellular stressed conditions, p53 is modified by ISG15 to enhance its transactivation function (49). To confirm the requirement for ISGylation in the clearance of misfolded dominant negative p53, we depleted myeloid cells of ISG15 and checked for the aggregated amyloid fibrils of p53 (Fig. 5). Our data support the model that ISG15 conjugates to nascent misfolded dominant negative p53 and mediates its degradation by the proteasome (Fig. 3 and 5).

Human ISG15 and USP18 are also induced by HIV-1 infection, type I IFNs, and genotoxic stress (18, 19, 24, 46, 57–59, 65, 67, 69). The sequential or parallel induction and expression of ISG15, USP18, and p53 in response to these stimulants are likely not due to chance but probably reflect a feedback regulatory mechanism between these proteins. Indeed, it is shown that *ISG15* is a downstream target gene of p53 (49), and the expression of ISG15 is likely required for the degradation of nascent misfolded p53 (46, 48). The stable expression of USP18 by lentiviral transduction of myeloid THP-1 cells induces a strong accumulation of p53 that appears dysfunctional for driving p21 mRNA and protein synthesis in differentiated THP-1 cells. This accumulated p53 exhibits a phenotype that is characteristic of misfolded dominant negative p53 (46, 76). Upon expression of active-site mutants of USP18, p53 did not accumulate, suggesting that the misfolded proteins were targeted for proteasomal degradation by ISG15-mediated modification. In contrast, the presence of wild-type USP18 abrogated the ISG15-mediated degradation of the misfolded p53, which apparently had the ability to inactivate the wild-type p53 function, as evidenced by decreased p21 mRNA and protein levels. Indeed, it is known that p53 mutants can form prion-like amyloid structures that accumulate in cells, which promote the wild-type p53 to adopt conformational changes that inactivate its function and are propagated in a prion-like manner (73, 77, 78, 80–82, 84). Thus, the conjugation of p53 to ISG15 could further prevent functional p53 from incorporating into aggregates and thereby help to preserve its transactivation function.

THP-1 cells possess a 26-bp deletion in exon 5 of one allele of *TP53*, which leads to a frameshift that introduces an early stop codon, so that this allele translates into an approximately 25-kDa protein with no suggested activity (110). The other allele appears intact with no alterations and translates into a 53-kDa protein. However, in the presence of USP18, p53 in the differentiated SAMHD1KO THP-1.USP18 cells failed to transactivate p21 mRNA and protein synthesis, implicating it as a misfolded dominant negative prion-like aggregate. Interestingly, overexpression of the 25-kDa mutant p53 protein in the THP-1 cells elevated the expression of the 53-kDa protein (Fig. 6C).

It is not clear which factor initially signaled p53 transcription and translation in the myeloid THP-1 cells leading to its accumulation in response to USP18 in the differentiated THP-1 cells. However, it is tempting to speculate that the transduction of the cells using lentiviral vectors may have activated the DNA damage response genes, including DNA-PK and ATM, following integration of the lentiviral vectors into the cell genome. Indeed, the time course of p53 induction following transduction with HIV-1 differed between THP-1 and BlaER1 cells, which have an intact p53 gene (Fig. 6A and B). The induction of p53 correlated with high expression of p21 and even more robustly in the presence of VPX in the transdifferentiated BlaER1 cells, suggesting that the absence of SAMHD1 via VPX-mediated degradation intensifies the extent of p53 stimulation possibly by DNA-PK or ATM and most probably by IFN stimulation following recognition and sensing of viral reverse transcripts (19, 51, 86–90, 93, 95, 111, 112). Thus, the newly synthesized misfolded p53 proteins in empty vector controls and the active-site mutant USP18 cells might have been cleared by the ISG15-mediated proteasomal degradation. However, USP18 by its protease activity retained accumulated misfolded dominant negative p53.

By regulating p21 transcription, p53 indirectly affects the replication of lentiviruses that requires dividing cells with large pools of dNTPs and inactive SAMHD1 as well as highly phosphorylated reverse transcriptase enzymes (13, 14, 16, 17, 27, 29, 35, 46–51, 53–56). It was recently shown that p53 and its downstream effector p21 block early and late stages of HIV-1 replication in monocyte-derived macrophages (MDMs) (13, 14, 56). Thus, the inability of the accumulated p53 proteins in the SAMHD1KO THP-1.USP18 cells to transactivate p21 mRNA and protein synthesis relieved p21 of its antiviral function, thereby enlarging the intracellular dNTP pool through activation of *de novo* dNTP biosynthesis, which allowed increased replication of HIV-1. Furthermore, many mutants of p53 have been shown to promote HIV-1 replication by activating HIV-LTR driven transcription (113). Consistent with this idea, DNA-binding domain inactive p53 mutant (R273H) as well as C-terminal truncated mutants of p53 supported high HIV-1 replication in differentiated THP-1 cells, while the wild type and the RD deletion mutant reduced HIV-1 replication.

Overall, we provide evidence that ISG15 and USP18 are factors that significantly contribute to HIV-1 infection in innate myeloid THP-1 cells by affecting misfolded p53 accumulation and relieving p21 of its inhibitory function, underlining that ISG15 conjugation to p53 is important for the *in vivo* clearance of misfolded dominant negative p53. Further work to explore p53-independent enhancement of HIV-1 replication by USP18 and how USP18 might modulate innate immune recognition is warranted, especially in primary cells, since our data were generated primarily in myeloid lineage cell lines.

## MATERIALS AND METHODS

### Plasmids.

Lentiviral pLOC vector (pEV) containing the open reading frames (ORFs) for the human wild-type *USP18* gene and its active-site mutants (C64A and C64S) were generated as described before (69). ISG15 plasmid was obtained from Renate König. E1 (UBE1L) and E2 (UBCH8) plasmids were kind gifts from Klaus-Peter Knobeloch. The human p53 ORF and its single-amino-acid mutant (R273H), cloned into plasmid pBC12 (114), were amplified and cloned into pLOC using the SpeI and AscI restriction sites. HA- and V5-tagged p53 and its R273H mutant were cloned into the same vector using the same restriction sites. p53 RDΔTp53 and CΔTp53 mutants were amplified from cDNAs, which were synthesized from genomic RNAs from THP-1 cells. Forward primer 5′-GTGACACGCTTCCCTGGAT and reverse primer 5′-GAGTTCCAAGGCCTCATTCA were used for the PCR amplification of RDΔTp53, and forward primer 5′-GTGACACGCTTCCCTGGAT and the reverse primer 5′-GAGTTCCAAGGCCTCATTCA were used for CΔTp53 amplification. The PCR amplicons were cloned into pJET1.2/blunt Cloning Vector (CloneJET PCR Cloning kit, K1232; Thermo Fisher Scientific, Karlsruhe, Germany), tagged with HA and V5 epitopes, and then sequenced. Multiple sequence alignments were performed, and protein expressions were confirmed by immunoblotting after sequencing. pSIN.PPT.CMV.Luc.IRES.GFP, pMDLg/pRRE, pMDLx/pRRE, HIV-2 VPX, pRSV-Rev, and pMD.G plasmids have been described before (69). The HIV-1 construct psPAX2 was obtained from the NIH, AIDS Reagent Program repository. pLentiCRISPRv2 plasmids targeting *ISG15* were constructed as described before (69, 115, 116). Briefly, complementary oligonucleotides, which contain the specific ISG15 single-guide RNA (sgRNA) sequences targeting different exons of the *ISG15* gene, including CACAGCCCACAGCCATGGTA for exon 1 (E1), ATCCTGGTGAGGAATAACAA for exon 2 (E2), and TTCCTCACCAGGATGCTCAG for exon 2 (E2b), and overhangs complementary to the overhangs generated by BsmBI digestion of the pLentiCRISPRv2 were ligated into the BsmB1-digested pLentiCRISPRv2 plasmid to generate a functional transfer vector. The pLentiCRISPRv2 plasmid, which lacked the sgRNA sequence, was used as a vector control.

### Cell culture.

Wild-type THP-1 cells (ATCC TIB-202) (117) and SAMHD1KO THP-1 cells (38) expressing pEV, USP18, and its mutants (C64A and C64S) were generated as described before (69). The human B cell precursor leukemia cell line, BlaER1, was obtained as a kind gift from Thomas Graf (118). ISG15KO BlaER1 and THP-1 cells were generated by HIV-1-based lentiviral transduction of these cells using purified particles containing the sgRNA guide transfer vector. Seventy-two hours postransduction, the cells were subcultivated in fresh medium containing 2 μg/ml of puromycin for selection over a period of 14 days. The selected cells were tested for the gene KO by immunoblotting for ISG15 expression and were subsequently used for experiments. All the BlaER1 and THP-1 cell lines were maintained in RPMI 1640 (PAN-Biotech, Aidenbach, Germany) supplemented with 10% fetal bovine serum (FBS), 2 mM l-glutamine, and 100 U/ml penicillin-streptomycin. The SAMHD1KO THP-1.USP18 strain and its mutants and pEV were supplemented with 2 μg/ml of blasticidin S hydrochloride (Sigma-Aldrich, Taufkirchen, Germany). HEK293T (ATCC CRL-3216) (119, 120) and 293A (R70507; Invitrogen, Karlsruhe, Germany) cells were maintained in Dulbecco’s modified Eagle’s medium (Biochrom, Berlin, Germany) supplemented with 10% FBS, 2 mM l-glutamine, and 100 U/ml penicillin-streptomycin. All cell lines were kept at 37°C in a humidified atmosphere with 5% CO_2_. The BlaER1 cells were transdifferentiated into monocytes as previously described (121). THP-1 cell lines were differentiated into macrophage-like cells as described previously (69). Peripheral blood mononuclear cells (PBMCs) were extracted by centrifugation of whole blood on a Ficoll/Hypaque density gradient (Biocoll separating solution; Biochrom AG). The isolated PBMCs were washed in Dulbecco’s phosphate-buffered saline, resuspended in RPMI medium supplemented with 10% FBS, 2 mM l-glutamine, and 100 U/ml penicillin-streptomycin, and stored at 37°C in a humidified atmosphere with 5% CO_2_. Next, 1 × 10^6^ cells were seeded in 6-well plates, stimulated with 1,000 μg/ml of phytohemagglutinin (PHA; Sigma-Aldrich) and 30 U/ml of interleukin 2 (IL-2; Sigma-Aldrich) for 3 days, and subsequently used for experiments. Activated PBMCs, SAMHD1KO THP-1.USP18 cells, or pEV were treated with MG132 (5 μM) (474790; Calbiochem), bafilomycin A1 (BafA1) (10 nM) (J61835; Alfa Aesar, Thermo Fisher Scientific, Karlsruhe, Germany), or dimethyl sulfoxide (DMSO) (A3672.0250; PanReac AppliChem, Darmstadt, Germany) for 16 h.

### Virus production and transduction.

HIV-1 luciferase reporter viruses were generated as described before (69). HIV-1 pseudotype virus containing the pLentiCRISPRv2 transfer vector, packaging plasmid vector psPAXs, and VSV-G were cotransfected into HEK293T cells. At 48 h posttransfection, viral supernatants were harvested, purified, concentrated over a 20% (wt/vol) sucrose cushion, resuspended in fresh RPMI medium, and stored in −80°C. All transduction assays were conducted by spinoculation at 30°C for 2 h at 1,200 × *g*.

### Quantitative RT-PCR.

Total genomic RNA from PMA-differentiated wild-type and SAMHD1KO THP-1.USP18 cells or pEV was isolated using a Qiagen RNeasy minikit (74106; Qiagen, Hilden Germany). One microgram RNA was transcribed into cDNA using RevertAid H-Minus First Strand cDNA Synthesis kit (K1631; Thermo Fisher Scientific Inc.). Reverse transcriptase PCR (RT-PCR) amplification of p21, p53, and as a control, HPRT1 (hypoxanthine guanine phosphoribosyltransferase) transcripts was performed using 1× Sybr green PCR master mix (4309155) using the ViiA7 Real-Time PCR system (Applied Biosciences, Thermo Fisher Inc.). The data were analyzed using the comparative threshold cycle (*C_T_*) mean and normalized to HPRT1. The following primers were used: for the amplification of p21 transcripts, forward primer 5′-TGGAGACTCTCAGGGTCGAAA and reverse primer, 5′-GGCGTTTGGAGTGGTAGAAATC; for p53 transcripts, forward primer 5′-GCCCAACAACACCAGCTCCT and reverse primer 5′-CCTGGGCATCCTTGAGTTCC; for HPRT1 transcripts, forward primer 5′-TGCTGAGGATTTGGAAAGGGT and reverse primer 5′-GGGCTACAATGTGATGGCCT.

### Transfection.

HEK293T cells were transfected with expression plasmids for V5-tagged wild-type p53, R273H, and RDΔTp53 constructs and untagged CΔTp53 mutants of p53 in 6-well plates using Lipofectamine LTX (Thermo Fisher Scientific Inc.). Forty-eight hours posttransfection, the cells were harvested and immunoblotted for V5 or p53 expression.

### Nondenaturing gel electrophoresis.

PMA-differentiated SAMHD1KO THP-1.pEV, USP18, and the C64A mutant cells, transdifferentiated ISG15KO BlaER1 and its pLV, and PMA-differentiated ISG15KO THP-1 and its pLV were lysed in mild lysis buffer on ice for 10 min. Supernatants were collected after lysates were cleared by centrifugation. Proteins were mixed with a sample buffer containing nondenaturing reagents, separated by nondenaturing gel electrophoresis, and immunoblotted for high-molecular-weight (HMW) p53 and amyloid fibrils using anti-p53 antibody and anti-amyloid antibody (OC) as described (76).

### Immunoblot analysis.

Cells were either lysed in radioimmunoprecipitation assay (RIPA) buffer as described before (69) or mild lysis buffer (50 mM Tris-HCl [pH 8], 150 mM NaCl, 0.8% NP-40, 10% glycerol, 1 mM phenylmethanesulfonyl fluoride solution [Sigma-Aldrich], and a tablet of protease inhibitor cocktail set III [Calbiochem]) for protein extraction. The following anti-human antibodies were used in this study: rabbit anti-USP18 (D4E7; Cell Signaling, Frankfurt, Germany [1:1000]), rabbit anti-SAMHD1 (12586-1-AP; Proteintech, Manchester, United Kingdom [1:1,000]), rabbit anti-p21 (2947.12D1; Cell Signaling [1:1,000]), goat anti-CDK2 (sc-163-G, M2; Santa Cruz Biotechnology [1:500]), rabbit anti-phospho-CDK2 (2561; Cell Signaling [1:1,000]), mouse anti-p53 (Ab-6, DO-1, OP43; Oncogene [1:500]) or mouse anti-p53 (Ab-3, PAb240, OP29; Oncogene [1:500]), rabbit anti-RRM2 (62-363; ProSci Biocat, Heidelberg, Germany [1:1,000]), mouse anti-TYMS (MAB4130, clone TS106; Merck, Darmstadt, Germany [1:500]), mouse anti-E2F1 (KH95, sc-251; Santa Cruz Biotechnology, Inc. [1:500]), rabbit anti-ISG15 (15981-1-AP; Proteintech [1: 500]), rabbit anti-HERC5 (22692-1-AP; Proteintech [1:500]), rabbit anti-EFP (TRIM25, 12573-1-AP; Proteintech [1:500]), rabbit anti-amyloid fibrils (OC) (AB2286; EMD Millipore, Darmstadt, Germany [1:500]), mouse anti-tubulin (clone B5-1-2; Sigma-Aldrich [1:7,500]), and goat anti-GAPDH (EB06377; Everest Biotech, VWR, Darmstadt, Germany [1:10,000]). Horseradish peroxidase (HRP)-conjugated sheep anti-mouse (α-mouse-IgG-HRP; GE Healthcare, Munich, Germany), donkey anti-rabbit (α-rabbit-IgG-HRP; GE Healthcare), and rabbit anti-goat (α-rabbit-IgG-HRP; Santa Cruz Biotechnology) antibodies were used as secondary antibodies, and blots were developed with ECL chemiluminescence reagents (GE Healthcare).

### Immunoprecipitation.

293A cells transfected with plasmids for hemagglutinin (HA)-tagged p53 (p53-HA), p53-HA and USP18, or p53-HA and active-site mutants of USP18 (C64A or C64S) in the presence of ISG15 and its conjugating enzymes E1 (UBE1L) and E2 (UBCH8) were harvested and lysed in mild lysis buffer. Proteins were subsequently cleared by centrifugation. The supernatants were incubated with 20 μl of anti-HA affinity matrix (Roche) at 4°C for gentle rotation overnight. The samples were washed six times with mild lysis buffer on ice. Bound proteins were eluted by boiling the beads for 5 min at 95°C in reducing reagent. The samples were subsequently immunoblotted for free ISG15 and its conjugates, HA, and USP18 using respective antibodies.

### Statistical analysis.

Data were analyzed using GraphPad Prism version 6 (GraphPad Software Inc., La Jolla, CA, USA). The study groups were compared using two-tailed unpaired Student's *t* tests, and a *P* value of <0.05 was considered statistically significant. Data are presented as means ± standard deviations (SDs).

### Ethical approval.

The blood bank of the Heinrich-Heine-University Hospital, Düsseldorf, Germany, provided buffy coats from anonymous blood donors after the ethics committee of the Medical Faculty of the Heinrich-Heine-University Düsseldorf (reference number 4767R-2014072657) approved the use of these samples for the study.
